# Association Between BDNF Val66Met Polymorphism and Mild Behavioral Impairment in Patients With Parkinson's Disease

**DOI:** 10.3389/fneur.2020.587992

**Published:** 2021-01-14

**Authors:** Mehrafarin Ramezani, Jennifer A. Ruskey, Kristina Martens, Mekale Kibreab, Zainul Javer, Iris Kathol, Tracy Hammer, Jenelle Cheetham, Etienne Leveille, Davide Martino, Justyna R. Sarna, Ziv Gan-Or, Gerald Pfeffer, Zahinoor Ismail, Oury Monchi

**Affiliations:** ^1^Department of Clinical Neuroscience, Hotchkiss Brain Institute, University of Calgary, Calgary, AB, Canada; ^2^Montreal Neurological Institute, McGill University, Montreal, QC, Canada; ^3^Department of Neurology and Neurosurgery, McGill University, Montreal, QC, Canada; ^4^Department of Human Genetics, McGill University, Montreal, QC, Canada

**Keywords:** BDNF, Parkinson's disease, mild behavioral impairment, neuropsychiatric symptoms, depression

## Abstract

Neuropsychiatric symptoms (NPS) are common in Parkinson's disease (PD) and have demonstrated an association with the p. Val66Met, a polymorphism in the *BDNF* gene. Mild behavioral impairment (MBI) is a validated syndrome describing emergent and persistent NPS in older adults as a marker of potential cognitive decline and dementia. This study investigated if PD patients with the Met allele were more likely to have MBI and whether they had impairments in specific domains of MBI using the Mild Behavioral Impairment Checklist (MBI-C) as the MBI ascertainment tool. One hundred forty-six PD patients were screened for neuropsychiatric and cognitive impairments with the MBI-C and the Montreal Cognitive Assessment (MoCA). All participants were genotyped for the *BDNF* p.Val66Met single-nucleotide polymorphism (SNP) using TaqMan Genotyping Assay. Statistical analysis was performed using multiple linear and logistic regression models. Met carriers had a 2 times higher likelihood of being MBI positive (MBI-C total score ≥8) than Val carriers. Met carriers had significantly higher MBI-C total scores and significantly greater impairments in the mood/anxiety and the psychotic domains of MBI-C compared to Val carriers. These findings indicate that the *BDNF* Met allele is associated with a higher neuropsychiatric burden in PD.

## Introduction

Neuropsychiatric symptoms (NPS) are common in Parkinson's disease (PD) patients. These NPS include depression, anxiety, psychosis, etc., which occur more frequently in PD patients than in the general population (1, 2). NPS can be present at early stages of PD and even precede the emergence of cardinal motor symptoms of PD (1). They have a severe social and emotional impact on the quality of life in PD patients and their families/caregivers (3). Mild behavioral impairment (MBI) is a validated syndrome characterized by the emergence of persistent NPS in older adults as an at-risk state for incident cognitive decline, and for some, MBI is the index manifestation of dementia, emerging in advance of cognitive symptoms (4). Early evidence in PD has linked MBI to altered corticostriatal connectivity, middle temporal lobe atrophy, and cognitive impairment which suggest a higher risk of developing dementia (5, 6).

Brain-derived neurotrophic factor (BDNF) is a crucial protein in the central nervous system (CNS) with a substantial role in differentiation, survival, and protection of CNS neurons (7). Studies have investigated a potential role for p.Val66Met (G758A, rs6265), a single-nucleotide polymorphism (SNP) in exon 11 of the *BDNF* gene, and PD (8–10). The p.Val66Met SNP substitutes a valine (Val) residue at position 66 with a methionine (Met) residue in the pro-domain of the BDNF protein (7).The Met allele has been found to be associated with cognitive impairments in PD patients and late-life psychiatric symptoms in the general population (8, 9, 11). This substitution is not transferred to the final form of BDNF; however, this structural change in the BDNF protein precursor can significantly decrease the secretion of BDNF extracellularly and subsequently reduce its availability to the CNS neurons (7). Recent evidence suggests a role of the Met allele in the induction of long-term depression (LTD) in the brain, likely via altered interaction of BDNF pro-domain with sortilin receptors (12, 13). The altered interaction might explain the connection of this polymorphism with NPS in the general population and in neurodegenerative diseases (14, 15). Recent longitudinal data in Alzheimer disease (AD) patients revealed strong evidence of Met association with depression (15). Moreover, recent meta-analysis reported higher likelihood of mild cognitive impairment in PD patients with the Met allele (9). These evidences suggest a link between the p.Val66Met polymorphism and NPS in PD patients and therefore encourage an investigation of this relationship.

In this study, we tested whether the p.Val66Met SNP in PD patients is associated with MBI burden using the Mild Behavioral Impairment Checklist (MBI-C). Specifically, we hypothesized that PD patients with at least one Met allele (Met carriers) would have greater likelihood of having MBI and higher total MBI-C score than those who are Val homozygotes (Val group). Additionally, we hypothesized that Met carriers would have higher MBI-C domain scores compared to Val group.

## Methods

### Participants

One hundred forty-six PD patients at Hoehn and Yahr stages II–III were recruited. Patients had a confirmed diagnosis of idiopathic PD by a Movement Disorder Clinic neurologist, meeting the UK Brain Bank criteria for idiopathic PD. All patients were on prescribed dopaminergic medication and were responsive to it. Exclusion criteria were the following: (1) any neurological disorder other than PD; (2) alcohol dependency; (3) history or presence of a severe psychiatric disorder; and (4) cerebrovascular disorders. The severity of motor symptoms was assessed using the motor section of the Unified Parkinson's Disease Rating Scale (UPDRS-III). All participants provided written informed consent according to the declaration of Helsinki, and the study was approved by the Conjoint Health Research Ethics Board (REB14-2463) at the University of Calgary.

### Genotyping

A blood sample was collected from each participant, and DNA was extracted using the MagMax DNA Multi-Sample Ultra 2.0 kit and the King Fisher Duo Prime Robot (Thermo Fisher Scientific). DNA samples were screened for the *BDNF* p.Val66Met SNP (rs6265) using TaqMan SNP Genotyping Assay C-11592758-10 on C-1000 Touch Thermal cycler (Bio-Rad). TaqMan assay reading was done on Applied Biosystems QuantStudio 7 Flex Real-Time PCR system (Fisher Scientific) according to the manufacturer's instructions. The TaqMan assay results were analyzed using the Bio-Rad CFX Maestro software.

### Neuropsychiatric and Cognitive Assessment

NPS in all participants was evaluated using the MBI-C (16, 17). The MBI-C contains 34 questions to cover the five domains of MBI including the following: (1) impaired drive/motivation (apathy); (2) emotional dysregulation (mood and anxiety symptoms); (3) impulse dyscontrol (agitation, aggression, abnormal reinforcement, and reward salience); (4) social inappropriateness (impaired social cognition); and (5) abnormal thoughts/perception (psychotic symptoms). This checklist is completed by each patient's caregiver/close family member. Consistent with the MBI criteria, symptoms should have lasted for at least 6 months and present a meaningful change of behavior from longstanding patterns. An MBI-C total score cut point of ≥8 was used to classify a patient as MBI case positive (5, 18, 19). All participants completed the Montreal Cognitive Assessment (MoCA) for a brief cognitive assessment and completed a questionnaire on their demographics and daily activity level.

### Statistical Analysis

Statistical analyses of continuous variables were performed using either the student *T* test or Mann-Whitney (M-W) *U* test based on the data normality. The Fisher exact and chi-square tests were used to test the categorical variables. Logistic regression was used to test the relationship between the MBI positive condition (the categorical dependent variable) and the two BDNF genotype groups, including any independent variables that were significantly different between the two conditions.

MBI-C total score (the continuous dependent variable) was compared between the two groups using a multiple linear regression model after checking for the multiple linear regression model assumptions. The independent variables that were correlated with MBI-C total score were included in the regression model. Values of *p* < 0.05 were considered significant for single tests, and significance of 0.01 was used to test MBI-C domains using Bonferroni correction. The same analysis was used to study association of p.Val66Met and MBI-C domain scores. All statistical tests were performed using IBM SPSS Statistics for Mac v. 26 (IBM Corp., Armonk, N.Y., USA). Power analysis was performed using G-Power software 3.1.9.6 (20).

## Results

### Demographics of Participants

The demographic and clinical characteristics of the participants are summarized in Table 1. Ten patients were identified as outliers based on values that were more than three standard deviation away from the mean of each allelic group for the following variables: age, education, UPDRS, Levodopa equivalent daily dosage (LEDD), disease duration, MoCA, and MBI-C total score.

**Table 1:** Demographic and clinical characteristics of PD participants.

**Characteristics mean, SD (Min–Max)**	**Val carriers (GG) *n* = 90**	**Met carriers (GA, AA) *n* = 46 (TT = 4)**	***p*-value**
**Age**	69.2, ±8.1 (47–86)	66.7, ±7.8 (48–79)	0.09^a^
**Sex (female percentage)**	36%	48%	0.20^b^
**Education (year)**	14.8, ±2.8 (8–21)	14.87, ±2.5 (9–19)	0.89^a^
**LEDD**	809.7, ±401.6 (200–1925)	822.3, ±373.3 (225–1675)	0.86^a^
**Disease duration (year)**	5.71, ±4.4 (0.2–16.1)	5.57, ±3.9 (0.2–18.2)	0.86^a^
**UPDRS-III**	18.2, ±10.0 (0–50)	20.3, ±11.2 (0–49)	0.27^a^
**MoCA**	25.3, ±4.0 (13–30)	25.9, ±3.2 (18–30)	0.42^a^
**Handedness** **Right-handed** **Left-handed** **Ambidextrous** **NA**	84% 12% 2% 1%	87% 6% 2% 4%	0.49^c^
**Ethnicity %** **Caucasian** **Other** **NA**	86.7% 7.8% 5.5%	91.3% 4.0% 4.0%	0.7^c^
**Exercise (hours per week)**	5.8 ± 4.8 (0–28) ^d^	6.2 ± 4.5 (0–20)	0.8^c^

*Outliers were identified based on three standard deviations away from the mean values of demographic and clinical characteristics; 10 participants were removed as outliers*.

*Val, valine; Met, methionine; SD, standard deviation; Min, minimum; Max, maximum; LEDD, Levodopa equivalent daily dosage; UPDRS-III, unified Parkinson's disease rating scale part III; MoCA, montreal cognitive assessment*.

a *Student t test*.

b *Fisher exact test (two-sided)*.

c *Chi-square test (two-sided)*.

d *Data were not available for two of the participants in the Val group*.

Among the 136 remaining PD participants, Val homozygous patients (GG) represented the majority of the cohort (*n* = 90). Because of the low number of homozygous Met/Met patients (*n* = 4), all Met carriers were pooled as one group. Forty-six patients were heterozygous or homozygous for the Met allele (GA, AA) with a frequency of 0.18, which was in accordance with Hardy-Weinberg equilibrium. The two groups had no significant differences in any of their demographic or clinical characteristics, ethnicity, and weekly exercise level (Table 1).

### Association of Val66Met and MBI-C Score

Met carriers were twice as likely to be MBI positive than the Val carriers; 39% of Met carriers were MBI positive, whereas in the Val group only 20% of patients were MBI positive. The Met group had a significantly greater mean value for MBI-C total score than Val carriers (7.39 vs. 4.06, respectively). Two factors were included in the multiple logistic regression as independent variables in addition to the *BDNF* groups based on significant differences between the two groups in the Mann-Whitney *U* test; MoCA and UPDRS-III (*M-W U* = 1230.5, *p* = 0.005, and *M-W U* = 2441.5, *p* = 0.002, respectively). The logistic regression analysis revealed a significant contribution of the Met allele for the likelihood of being MBI positive (*OR* = 2.88, CI 95% = 1.22–6.78, *p* = 0.02) (Table 2).

**Table 2:** Multiple logistic regression analysis of *BDNF* p.Val66Met and MBI positive likelihood.

**Covariate**	**Estimate**	**SE**	**Wald's chi square**	*p*-value^*^	**OR**	**95% CI**
*BDNF* Met allele	1.06	0.44	5.82	0.02^a^^*^	2.88	1.22–6.78
MoCA	−0.14	0.06	5.96	0.02^a^^*^	0.87	0.78–0.97
UPDRS-III	0.05	0.02	4.68	0.03^a^^*^	1.05	1.00–1.09
Constant	0.10	1.60	0.004	0.95^a^	NA	NA

*Val, valine; Met, methionine; MBI-C, mild behavioral impairment checklist; SE, standard error; OR, odds ratio; CI, confidence interval; NA, not applicable*.

a *Multiple logistic regression model, N = 136, Nagelkerke pseudo R^2^ = 0.22, Hosmer and Lemeshow goodness-of-fit test p = 0.85 (df = 8), correct cases overall percentage = 74.3%*.

* *p < 0.05*.

**Figure 1:**
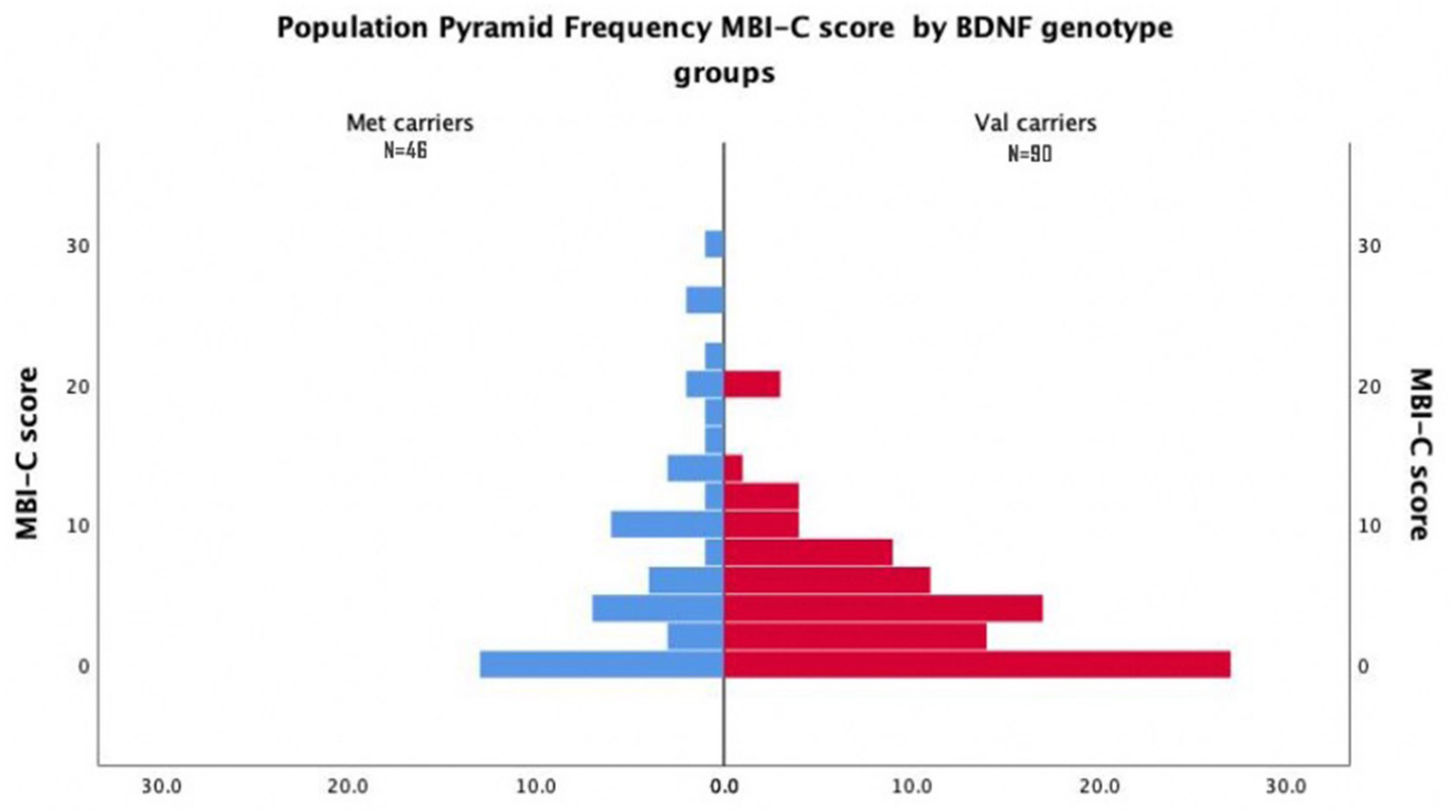
Population pyramid frequency MBI-C score by BDNF genotype groups.

Most Val Carriers had Either Zero or Very Low MBI-C Total Score (Figure 1).

MBI-C total and MoCA scores were negatively correlated [Pearson's *r* = (134) 0.17, *p* = 0.04]. Also, UPDRS-III and MBI-C total scores had a positive correlation [Pearson's *r* = (134) 0.23, *p* = 0.007]. These factors were included in the multiple linear regression model. The difference between the MBI-C total score between the two allelic groups was statistically significant when controlling for MoCA and UPDRS-III scores in the regression model [*r*^2^ = 0.13, *Beta* = 0.25, *F* (3, 135) = 6.36, *p* = 0.013, Cohen's *f* ^2^ = 0.15]. A power analysis was conducted, which revealed that our samples size of 136 would yield a power of 0.99 assuming type-I error rate of 0.05.

Association results of *BDNF* alleles with MBI-C domain scores are shown in Table 3. Patients with the Met allele had significantly higher MBI-C scores for the mood/anxiety (*r*^2^ = 0.10, *Beta* = 0.24, *p* = 0.004) and the psychosis domains (*r*^2^ = 0.12, *Beta* = 0.23, *p* = 0.006) when controlling for MoCA and UPDRS-III scores (Table 3).

**Table 3:** MBI-C total and domain scores.

**MBI-C scores Mean, SD (Min–Max)**	**Val carriers (GG) *N* = 90**	**Met carriers (GA, AA) *N* = 46**	***r*^**2**^, Beta (CI 95%)**	*p*-value^*^
MBI-C total	4.06, ±4.50 (0–20)	7.39, ±8.05 (0–29)	0.13, 0.25 (1.17–5.35)	0.003^**^
Drive/motivation	1.10, ±1.72 (0–8)	1.43, ±2.02 (0–8)	0.06, 0.07 (−0.38 to 0.92)	0.41
Mood/anxiety	1.48, ±2.21 (0–13)	2.87, ±3.36 (0–12)	0.10, 0.24 (0.46–2.35)	0.004^**^
Impulse dyscontrol	0.79, ±1.43 (0–8)	1.15, ±2.80 (0–13)	0.03, 0.10 (−0.28 to 1.16)	0.23
Social inappropriateness	0.16, ±0.62 (0–4)	0.43, ±1.41 (0–7)	0.04, 0.14 (−0.07 to 0.63)	0.11
Abnormal thoughts/perception	0.53, ±1.09 (0–4)	1.50, ±2.54 (0–11)	0.12, 0.23 (0.26–1.48)	0.006^**^

*MoCA and UPDRS scores were used in a multiple linear regression model. In total, 10 PD participants were identified as outliers and removed from the analysis*.

*Val, valine; Met, methionine; MBI-C, mild behavioral impairment checklist; SD, standard deviation; Min, minimum; Max, maximum; MoCA, montreal cognitive assessment; CI 95%, 95% confidence interval*.

* *Analysis was performed using multiple linear regression model including MoCA and UPDRS scores in the model. MoCA and MBI-C total scores were negatively correlated (r_p_ = 0.17, p = 0.043), while UPDRS and MBI-C total scores were positively correlated (r_p_ = 0.23, p = 0.007)*.

** *p-value was set to <0.01 to correct for multiple tests, Bonferroni correction*.

We performed an extra analysis in order to confirm that MBI classification results were derived from *BDNF* alleles and not driven by a few participants with marginally higher or lower MBI-C total score than the cutoff value (≥8). All participants with an MBI-C total score of 7 and 8 were excluded from the sample, and analysis was repeated. In total, 10 patients were removed; only one Met-carrier had the score of 7. In the Val group, three patients had the score of 7, and six patients had the score of 8 for MBI-C. Similar to the main analysis, two factors were included in the multiple logistic regression as independent variables in addition to the *BDNF* groups, based on their significant differences between the two groups in the Mann-Whitney *U* test; MoCA and UPDRS-III (*M-W U* = 1024.5, *p* = 0.02, and *M-W U* = 1914.5, *p* = 0.007, respectively). Results revealed a significant contribution of the Met allele for the likelihood of being MBI positive (*OR* = 4.38, CI 95% = 1.72–11.14, *p* = 0.002) (Table 4).

**Table 4:** Multiple logistic regression analysis of *BDNF* p.Val66Met and MBI positive likelihood after removing participants with total MBI-C score of 7 and 8.

**Covariate**	**Estimate**	**SE**	**Wald's chi square**	*p*-value^*^	**OR**	**95% CI**
*BDNF* Met allele	1.48	0.48	9.54	0.002^a^^*^	4.37	1.71–11.14
MoCA	−0.14	0.06	4.84	0.03^a^	0.87	0.77–0.98
UPDRS-III	0.04	0.02	3.76	0.053^a^	1.04	1.00–1.09
Constant	−0.71	1.71	0.19	0.67^a^	NA	NA

*Ten participants were removed, including only one Met carrier with the score of 7 (n = 126)*.

*Val, valine; Met, methionine; MBI-C, mild behavioral impairment checklist; SE, standard error; OR, odds ratio; CI, confidence interval; NA, not applicable*.

a *Multiple logistic regression model, N = 126, Nagelkerke pseudo R^2^ = 0.24, Hosmer and Lemeshow goodness-of-fit test p = 0.31 (df = 8), correct cases overall percentage = 77.8%*.

* *p-value was set to <0.01 corrected for multiple tests, Bonferroni correction*.

Each MBI-C domain score was compared between the two groups using Mann-Whitney *U* test (Table 5). The results were similar to the whole cohort analysis. Met carriers had significantly higher MBI-C total score than the Val group. Also, Met carriers had significantly higher score for the mood/anxiety domain when compared to the Val group and a trend for higher psychosis score (Table 5).

**Table 5:** MBI-C total and domain scores after removing participants with total MBI-C score of 7 and 8.

**MBI-C scores Mean, SD (Min–Max)**	**Val carriers (GG) *N* = 81**	**Met carriers (GA, AA) *N* = 45**	*p*-value^*^
MBI-C total	3.65, ±4.56 (0–20)	7.40, ±8.14 (0–29)	0.03^**^
Drive/motivation	1.00, ±1.72 (0–8)	1.40, ±2.03 (0–8)	0.26
Mood/anxiety	1.35, ±2.21 (0–13)	2.84, ±3.40 (0–12)	0.008^**^
Impulse dyscontrol	0.64, ±1.34 (0–8)	1.18, ±2.83 (0–13)	0.87
Social inappropriateness	0.10, ±0.46 (0–3)	0.44, ±1.42 (0–7)	0.09
Abnormal thoughts/perception	0.57, ±1.13 (0–4)	1.53, ±2.56 (0–11)	0.02

*Ten participants were removed, including only one Met carrier with the score of 7 (n = 126)*.

*Val, valine; Met, methionine; MBI-C, mild behavioral impairment checklist; MoCA, montreal cognitive assessment; SD, standard deviation; Min, minimum; Max, maximum*.

* *All the analysis was done by Mann–Whitney U test because of the data normality*.

** *p-value was set to <0.01 to correct for multiple tests, Bonferroni correction*.

## Discussion

To our knowledge, the present study is the first to explore the association of the *BDNF* p.Val66Met SNP and MBI in patients with PD. Patients with at least one Met allele had significantly higher MBI-C total score and significantly higher scores in the emotional dysregulation and the abnormal thoughts/perception domains. Furthermore, PD patients with at least one Met allele had a significantly higher prevalence of MBI than patients in the Val group using MBI-C as the case ascertainment instrument. Our findings implicate the *BDNF* p.Val66Met SNP in the pathogenesis of MBI in PD patients and suggest this variant as a genetic risk factor for MBI in PD with a medium effect size (Cohen's *f* ^2^ = 0.15). These findings are consistent with the evidence in the AD, which imply that the Met allele can be a risk factor for incident cognitive decline and dementia in PD (4, 15, 21).

An increasing body of evidence suggests a link between the development of NPS and cognitive decline in different types of dementia (5, 22–24). Studies demonstrating that *BDNF* p.Val66Met SNP is found to be associated with both NPS and cognitive impairments in AD are consistent with a biological understanding of NPS (25–29), and previous evidence linking *BDNF* and NPS (8, 15). The presence of amyloid-β pathology in PD patients together with Lewy body pathology might be a possible explanation of similar NPS profile in AD and PD patients. However, it should be mentioned that different studies have teased apart how the psychiatric profile of PD and AD patients are different (30, 31).

Patients who experience NPS in the early stages of PD show an increased risk of cognitive decline (1, 5), which is consistent with the findings in non-PD dementia (24, 32–36). A recent study reported that PD patients with a variety of NPS, for example, depression, apathy, and hallucinations, displayed impairments in at least one of the main cognitive domains (executive function, language, memory, attention, and visuospatial) and in global cognition (5). These findings hint at the importance of early diagnosis of sustained NPS as markers of cognitive decline in PD patients, in order to identify patients at risk of incident cognitive decline and dementia.

A recent meta-analysis reported an association between the *BDNF* Met allele and cognitive impairments in PD for 532 patients and 802 controls (*p* = 0.003). However, the cognitive impairments were found to be more specific to the Caucasian populations (9). Several studies suggested that p.Val66Met SNP might have an association with depression, particularly geriatric depression (14, 37, 38). Nonetheless, this association might differ based on a variety of factors, e.g., the origin of the study population, sex, and the fundamental issue of whether depression is chronic, recurrent, or of later life onset as most depression rating scales do not differentiate (8, 33). The association of *BDNF* Met allele and geriatric depression was investigated in a meta-analysis including 523 cases and 1,220 controls (age ≥ 60 years) (14). An association between the Met allele and an increased risk for late-life depression was reported (*p* = 0.004) (8). However, one study reported that the Val allele is associated with anxiety and depression using the Beck Depression Inventory (BDI) and Beck Anxiety Inventory (BAI) in 104 PD patients. Nevertheless, their population structure was greatly different than the one in our study. Seventeen percent of their participants had early onset PD with a positive family history (39), while in our sample there were only two participants (1.4%) with early onset PD and all of the participants had a confirmed diagnosis of idiopathic PD. Other reasons explaining the different results between the Cagni et al., (39) study and ours are linked to measurement differences of NPS. The BDI and BAI are self-report measures assessing the presence of mood and anxiety symptoms over the last 2 and 4 weeks, respectively. In contrast, the MBI-C measures later life emergent and persistent (for at least 6 months) NPS, identified by a reliable informant. These are quite different approaches to measurement of symptoms, with the MBI-C developed explicitly to capture later life emergent symptoms that either serve as risk factors for cognitive decline and dementia or more likely represent early manifestations of dementia.

Furthermore, BDNF involvement in depression/anxiety disorders has been confirmed through measuring peripheral BDNF levels as well (40, 41). A meta-analysis reported a strong evidence of an association between depression and a decrease in BDNF levels (*p* < 6.8 × 10^−8^) (40). These meta-analyses highlight the crucial role of BDNF in depressive disorders and specifically the impact of p.Val66Met SNP on geriatric depression. The Met allele exhibits LTD properties, reduces the neural plasticity, and can also substantially affect the docking of BDNF secretory vesicles into the cellular membrane and decrease its release into the synaptic cleft (7). The BDNF pro-domain with a Met residue is shown to have an independent function by induction of LTD, reducing spine density and neuronal plasticity. These molecular changes are linked to depression and anxiety disorders in both animal models and clinical studies (7, 42, 43). These findings are in agreement with our results that PD patients with at least one Met allele are more susceptible to impairments in the affective/mood dysregulation and abnormal thoughts/perception domains of MBI-C.

We found a strong association between abnormal thoughts/perception in PD patients and Met allele in our cohort. Abnormal thoughts/perception represent psychotic symptoms, specifically hallucinations and delusions, which are associated with impairments in global cognition (1, 44). An abrupt visual memory function is suggested as a potential cause of visual hallucinations. Since BDNF plays a prominent role in the molecular mechanisms of memory in hippocampus, this indicates a possible role for BDNF in the development of such NPS (1, 7). Meta-analytical results of p.Val66Met SNP and psychotic disorders, for example, schizophrenia, are inconclusive at the moment (8, 45). However, the Met allele was found to be linked to higher susceptibility to hippocampal volume loss and deteriorated memory abilities in bipolar patients (46). These findings are in agreement with our results that PD patients with at least one Met allele are more susceptible to symptoms in the affective/mood dysregulation and abnormal thoughts/perception domains.

This study has some limitations that need to be considered. Although the findings of this study showed a fair level of robustness, the sample size is relatively small. The results of this study need to be replicated in a larger sample with an age-matched control group. Nevertheless, the *post hoc* power analysis indicates sufficient power in our cohort to detect the true effect of *BDNF* p.Val66Met. A full cognitive assessment of participants would benefit the exploration of p.Val66Met SNP impact on the aging brain, specifically in PD. Antidepressant medications was not considered. It has been shown that antidepressant medications can elevate peripheral BDNF and improve reversible NPS (40).

In conclusion, we observed an association between the *BDNF* p.Val66Met SNP and susceptibility for the development of late-life behavioral changes in PD patients. PD patients with at least one Met allele had a higher likelihood of MBI compared to non-carriers. Moreover, PD patients with one Met allele had a greater tendency to exhibit mood and anxiety symptoms as well as psychotic symptoms compared to the Val carriers. These findings indicate a potential role for the *BDNF* p.Val66Met SNP in late-life psychiatric impairment, subsequent cognitive decline, and dementia in PD patients.

## Data Availability Statement

The data that support the finding of this article are available from the corresponding author, upon request. Requests to access the datasets should be directed to Oury Monchi, Monchioury.monchi@ucalgary.ca.

## Ethics Statement

The studies involving human participants were reviewed and approved by Conjoint Health Research Ethics Board (REB14-2463) at the University of Calgary. The patients/participants provided their written informed consent to participate in this study.

## Author Contributions

MR: study design, data analysis, interpretation, manuscript writing, and revision. JR, KM, MK, ZJ, IK, TH, JC, EL, DM, JS, ZG-O, and GP: data collection. ZI: study design, analysis, and manuscript revision. OM: study design, data analysis, supervision, and manuscript revision. All authors contributed to the article and approved the submitted version.

## Conflict of Interest

The authors declare that the research was conducted in the absence of any commercial or financial relationships that could be construed as a potential conflict of interest.
